# Neuroimaging Markers of Mal de Débarquement Syndrome

**DOI:** 10.3389/fneur.2021.636224

**Published:** 2021-03-04

**Authors:** Yoon Hee Cha, Lei Ding, Han Yuan

**Affiliations:** ^1^Department of Neurology, University of Minnesota, Minneapolis, MN, United States; ^2^Stephenson School of Biomedical Engineering, University of Oklahoma, Norman, OK, United States; ^3^Institute for Biomedical Engineering, Science, and Technology, University of Oklahoma, Norman, OK, United States

**Keywords:** mal de débarquement syndrome, persistent oscillating vertigo, functional MRI, voxel-based morphometry, positron emission tomography, independent component phase coherence, noninvasive brain stimulation

## Abstract

Mal de débarquement syndrome (MdDS) is a motion-induced disorder of oscillating vertigo that persists after the motion has ceased. The neuroimaging characteristics of the MdDS brain state have been investigated with studies on brain metabolism, structure, functional connectivity, and measurements of synchronicity. Baseline metabolism and resting-state functional connectivity studies indicate that a limbic focus in the left entorhinal cortex and amygdala may be important in the pathology of MdDS, as these structures are hypermetabolic in MdDS and exhibit increased functional connectivity to posterior sensory processing areas and reduced connectivity to the frontal and temporal cortices. Both structures are tunable with periodic stimulation, with neurons in the entorhinal cortex required for spatial navigation, acting as a critical efferent pathway to the hippocampus, and sending and receiving projections from much of the neocortex. Voxel-based morphometry measurements have revealed volume differences between MdDS and healthy controls in hubs of multiple resting-state networks including the default mode, salience, and executive control networks. In particular, volume in the bilateral anterior cingulate cortices decreases and volume in the bilateral inferior frontal gyri/anterior insulas increases with longer duration of illness. Paired with noninvasive neuromodulation interventions, functional neuroimaging with functional magnetic resonance imaging (fMRI), electroencephalography (EEG), and simultaneous fMRI-EEG have shown changes in resting-state functional connectivity that correlate with symptom modulation, particularly in the posterior default mode network. Reduced parieto-occipital connectivity with the entorhinal cortex and reduced long-range fronto-parieto-occipital connectivity correlate with symptom improvement. Though there is a general theme of desynchronization correlating with reduced MdDS symptoms, the prediction of optimal stimulation parameters for noninvasive brain stimulation in individuals with MdDS remains a challenge due to the large parameter space. However, the pairing of functional neuroimaging and noninvasive brain stimulation can serve as a probe into the biological underpinnings of MdDS and iteratively lead to optimal parameter space identification.

## Introduction

Mal de débarquement syndrome (MdDS) is a disorder of persistent oscillating vertigo that follows entrainment to passive motion such as during water, air, or land travel (1). Symptoms are described as a “rocking,” “bobbing,” or “swaying” perception that is nulled by exposure to passive motion such as driving/riding in a car or returning to the triggering stimulus. The nonspinning vertigo of MdDS is not based on any underlying peripheral vestibular dysfunction (2). It was hypothesized and then shown in neuroimaging studies using positron emission tomography (PET), structural magnetic resonance imaging (MRI), functional MRI (fMRI), electroencephalography (EEG), and simultaneous fMRI-EEG that there are central nervous system correlates of MdDS that are involved in the persistence of the disorder (3). These imaging correlates can be captured as baseline metrics and as dynamic changes following symptom modification with noninvasive brain stimulation. These studies indicate that functional connectivity measured by fMRI or EEG can be used as markers of MdDS and serve as guides for neuromodulation-based interventions.

### Positron Emission Tomography

**Figure 1:**
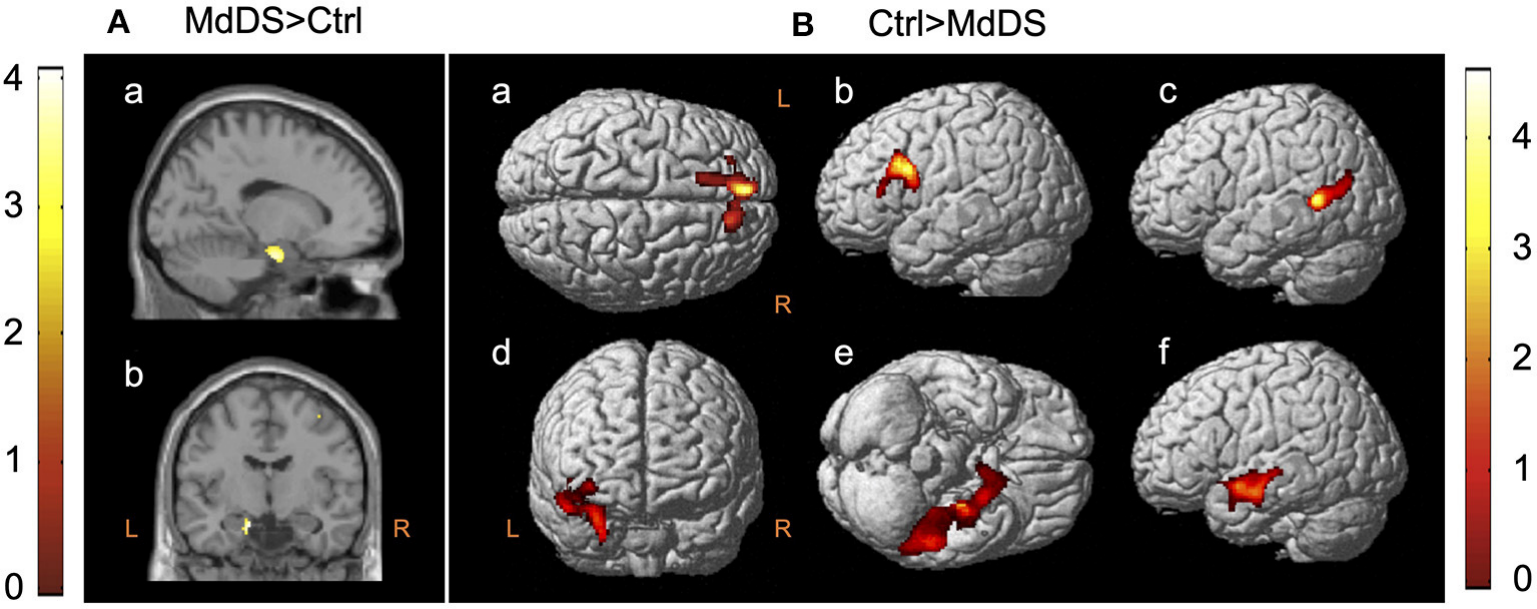
^18^F-FDG PET contrasts between mal de débarquement syndrome (MdDS) and controls (Ctrl) with differences of *z* > 3.3. Clusters are shown at *z* > 2.57 for the MdDS > Ctrl contrast and at *z* > 1.96 for the Ctrl > MdDS contrast for better visualization. Coordinates of peak significance are indicated in parentheses. In SPM, negative *X*-values are on the left and positive on the right. **(A)** MdDS > Ctrl: left entorhinal cortex/amygdala [Montreal Neurological Institute (MNI): −14, −8, −22], (a) sagittal view and (b) coronal view. **(B)** Ctrl > MdDS: (a) left superior medial gyrus (MNI: −8, 52, 36), (b) left middle frontal gyrus (MNI: −32, 18, 30), (c) left middle temporal gyrus (MNI: −50, −52, 8), (d) right insula/amygdala (MNI: 30, −2, −26 and 40, 2, −8), (e) left inferior temporal gyrus (MNI: −52, −38, −24), and (f) left superior temporal gyrus (MNI: −52, 0, −14). Figure adapted from Cha YH et al., *PLOS One* 2012 (4).

The earliest neuroimaging study and the only PET study that evaluated glucose metabolism during ongoing MdDS symptoms was reported by Cha et al. (4) in 2012. In this study, 20 individuals with MdDS (mean age 43.4 years, range 27–66 years) with a median symptom duration of 17.5 months (range 3–240 months) underwent ^18^F^18^-FDG PET and were compared with 20 age-, sex-, and handedness-matched healthy controls. These participants underwent structural and resting-state fMRI on the same day immediately before the PET scan. After age and gray matter volume were corrected, as well as concurrent measures of anxiety and depression, a single cluster of hypermetabolism (*z* > 3.3) at Montreal Neurological Institute (MNI) coordinates (−14, −8, −22) mapping to the junction between the left entorhinal cortex and amygdala was revealed (Figure 1A). Compared with the singular area of hypermetabolism in MdDS, there was a larger volume of cortex that exhibited hypometabolism. These areas were largely in the prefrontal and temporal cortices ipsilateral to the area of hypermetabolism (Figure 1B).

The potential role of the entorhinal cortex in MdDS pathophysiology discovered in this study relates to its function as a hub in a widespread brain network involved in memory, navigation, and mapping of self-location (5, 6). It provides the main efferent input into the hippocampus and is highly interconnected with the neocortex (5, 7, 8). Medial entorhinal cortex neurons fire in a hexagonally tuned manner sensitive to gaze direction, direction of heading, and speed (9–12). Graded persistent activity that is tunable to the periodicity of the stimulus is noted in both the entorhinal cortex and amygdala neurons, which is of particular relevance to MdDS since the main triggers of MdDS are characterized by oscillating motion inputs, and the entorhinal cortex is at least partially under vestibular influence (13–16). The functional consequences of reduced metabolism in the prefrontal and temporal areas are less clear, as all these areas are heteromodal, but both the left superior frontal gyrus and the left middle frontal gyrus just anterior to the precentral gyrus (as specifically found in this study) have been shown to activate during tasks of introspection and deactivate during sensorimotor processing (17).

While it may be premature to assign the functional relevance of the left-sided bias of the differential metabolic activity in this study to the semiology of MdDS, both the entorhinal cortex and the hippocampus to which it projects do exhibit some lateralization of function. Both the left and right entorhinal cortices activate during object recognition and spatial processing tasks, with the left side biased toward object recognition and the right side biased toward spatial processing (18). This is consistent with electrical recordings with subdural and depth electrodes in people doing virtual reality tasks in which low theta (1–3 Hz) power increases in the left hippocampus during semantic memory tasks but in the right hippocampus during spatial navigation tasks (19). Navigation studies in humans playing virtual reality games during fMRI acquisition indicate that the left hippocampus activates when the participant uses “egocentric,” self-orientation cues whereas the right hippocampus activates when they use “allocentric,” environmental cues as navigation strategies (20). These studies indicate that the left entorhinal cortex–hippocampus bias toward semantic processing may be used to navigate based on self-reference (e.g., the person goes to *their* “left, then right, then left”), whereas the right entorhinal cortex–hippocampus bias toward spatial processing may be used to navigate based on an awareness of an environmental map (e.g., the person navigates in relation to where they are in a map). As a hypothesis, a shift to a semantic memory-based egocentric strategy for navigation may be more useful when external sensory stimuli cannot be processed reliably.

One concern related to the discovery of a limbic focus of altered activity in MdDS is the potential confound of concurrent depression and anxiety. In order to determine whether these metabolism findings could be related to concurrent anxiety or depression, the anxiety and depression subscores of the Hospital Anxiety and Depression Scale (HADS) were used as nuisance regressors and were additionally used in multiple regression analyses to determine which brain regions correlated most strongly with these scores (21). Depression scores correlated with increased metabolism in the pregenual anterior cingulate cortex (pgACC), while anxiety scores correlated with increased metabolism in the dorsal midbrain and anterior temporal lobes. These regions did not show any overlap with regions that showed differential metabolism between MdDS and healthy controls but had been previously identified as brain regions related to mood and anxiety disorders (22–24). Interestingly, though activation of the amygdala is typical in fMRI studies on mood and anxiety disorders, metabolic abnormalities are not found in limbic areas in these disorders but rather in the prefrontal, pregenual, and basal ganglia (25–27). Only one study in depression found an increased amygdala metabolism localized to the right side (28).

A second PET study of relevance to MdDS compared baseline cerebral metabolic differences in fishermen who were prone to land sickness vs. those who were not (29). In this study of 28 fishermen, 15 were susceptible to developing transient symptoms of land sickness for 2–6 h after coming off of fishing expeditions, while 13 were not. All peripheral vestibular testing metrics including video-oculography, video head impulse test (vHIT), cervical vestibular evoked myogenic potentials (cVEMPs), and ocular VEMPS (oVEMPs) were normal in the two groups. Though there was a trend toward younger age in the group that tended to develop land sickness vs. those who did not (mean 50.9 vs. 56.7 years), there was no difference in terms of years of experience or time at sea between the two groups. However, there were two notable differences between individuals who tended to develop land sickness and those who did not: land sickness-prone individuals performed better on a visuospatial short-term memory test called the Corsi block test, and they were much less likely to suffer from motion sickness on other modes of transportation. These individuals, who were evenly split between men and women, were imaged during the nonsymptomatic period.

In the individuals who were prone to developing land sickness, there was hypometabolism in the right cerebellar inferior semilunar lobule (HVIIA), uvula, nodulus, and tonsil with relative hypermetabolism in the bilateral prefrontal and occipital cortices, specifically in the left superior occipital, superior, and inferior parietal lobules (SPL and IPL), and bilateral superior frontal gyri including the dorsolateral prefrontal cortex (DLPFC). The foci that survived a statistical threshold of *z* > 3.3 were the right superior frontal gyrus and the left SPL. Structural imaging was reported as normal, but areas of difference in metabolism were not corrected for differences in gray matter volume.

Reduced vestibulocerebellar metabolism in land sickness-prone individuals was postulated to relate to suppression of visual–vestibular inputs during continuous vestibular activation during wave-motion exposure, while higher metabolism in the occipital cortices in these individuals was postulated to relate to greater visual dependence with perhaps a heightened ability to suppress low-frequency vestibular signals that can trigger motion sickness. Similarly, higher prefrontal activity was postulated to be involved in the regulation of mood and anxiety. Along with the increases in metabolism in the SPL, this higher prefrontal activity may contribute to enhanced visual orientation and visuospatial attention required to suppress motion sickness but could potentially lead to more land sickness.

Given the inclusion of different sexes and symptom states, it is not possible to directly compare the results of these two PET studies. Land sickness is well-recognized as a common phenomenon affecting over 70% of otherwise healthy individuals and is not considered to be pathologic (30–32). Consistent with prior studies on land sickness, the PET study on land sickness-prone fishermen found a roughly equal distribution of men and women in the groups prone to land sickness and those who were not (33, 34). This is in contrast to studies on persistent MdDS that show an overwhelming predominance of women (3, 33). However, both studies contribute to an understanding of how brain metabolism signatures can represent both current symptoms and serve as markers of vulnerability to post-motion oscillating vertigo.

## Resting-State Functional Magnetic Resonance Imaging

Low-frequency spontaneous oscillations in cortical activity can be measured through blood oxygen level-dependent (BOLD) signals to reveal functionally connected regions (35, 36). Functional connectivity may be quantified by measurement of correlated activity between regions of interests or through independent component analysis to reveal intrinsic differences in connectivity between populations. Measurement of the synchronicity of very low-frequency BOLD fluctuations has revealed that the brain is organized into a number of functionally distinct but spatially distributed networks across the cerebrum and cerebellum (37, 38). Temporally correlated activity within these distributed regions defines a resting-state network with over a dozen networks that have been consistently identified across studies. While some networks are related to specific tasks such as auditory, visual, language, and sensorimotor, there are others that are localized to particular regions such as the basal ganglia, posterior insula, and precuneus (39). Three networks that are considered to be “amodal” include the default mode network (DMN), the salience network (SN), and the executive control network (ECN) (aka central executive network) (39). These networks are involved in the general tasks of shifting attention and processing of self-referential or externally driven mental activity and function across task-specific networks. Imaging studies indicate a role for each of these networks in MdDS pathophysiology. Of note, MdDS is significantly more common in women, but connectivity within these major amodal intrinsic brain networks does not appear to be the reason, as they are robustly detectable in men and women to a similar degree (40).

The DMN is activated during self-referential information processing and is composed of key nodes in the medial prefrontal cortex (mPFC), posterior cingulate cortex (PCC), precuneus, intraparietal sulcus (IPS), and hippocampal formation (HF+), which includes the entorhinal cortex (41, 42). The DMN connection to the hippocampus proper flows through the entorhinal cortex (43). These hubs play critical roles in memory retrieval, navigation, internal mental monitoring, and taking on the perspective of others (39, 42). Dysfunction within the DMN has been noted in a growing number of brain disorders including autism, Alzheimer's disease, depression, and schizophrenia (42, 44).

The SN has major hubs in the anterior insula and the anterior cingulate cortex (ACC) and serves to shift attention to behaviorally relevant stimuli whether internal or external (45). The ECN is anchored in the DLPFC, ventrolateral prefrontal cortex (VLPFC), and supramarginal gyrus and is involved in working memory and directed attention (39).

Resting-state fMRI data acquired in 20 individuals with MdDS and 20 healthy controls were used to calculate the degree of functional connectivity between the entorhinal cortex/amygdala area of hypermetabolism and specific areas of the neocortex (4). Pearson's correlation coefficients were calculated between the entorhinal cortex/amygdala seed and primary visual cortex V1, motion-sensitive area V5 (localized with a visual motion task), SPL, the frontal eye fields (FEF) (localized with a saccade task), and all the regions that were hypometabolic in MdDS individuals compared with controls.

**Figure 2:**
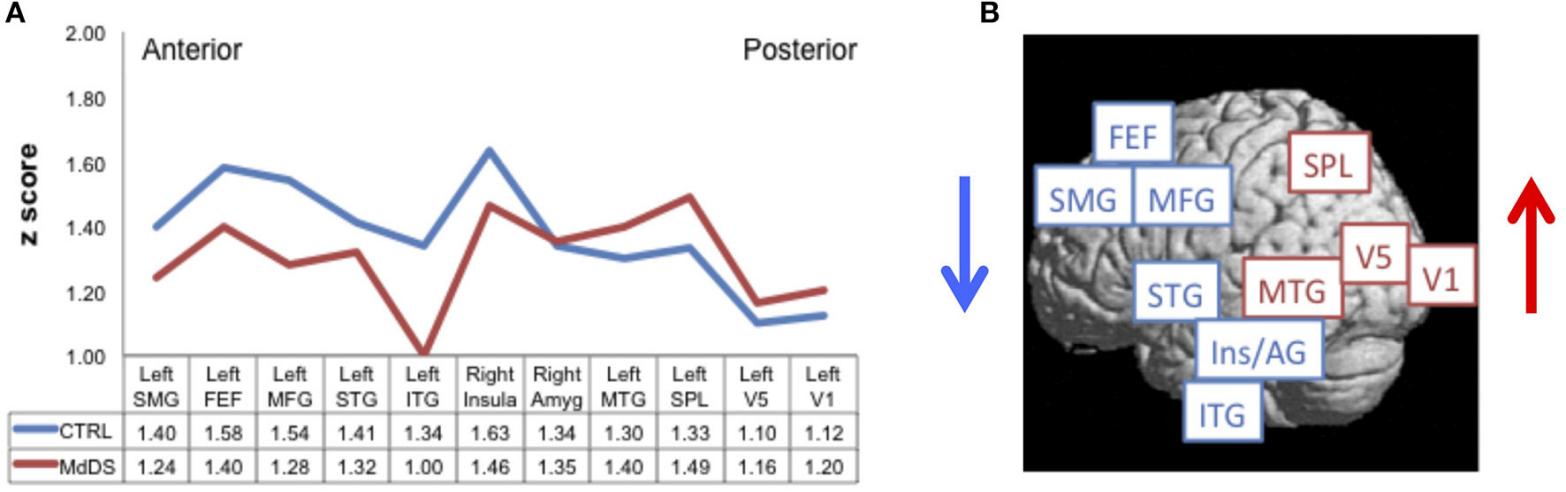
Resting-state functional connectivity reflected by Pearson correlation coefficients converted to *z*-scores **(A)** between the left entorhinal cortex/amygdala seed of hypermetabolism [entorhinal cortex/amygdala (EC/AG)] and regions of hypometabolism [left superior medial gyrus (SMG), left middle frontal gyrus (MFG), left superior temporal gyrus (STG), left inferior temporal gyrus (ITG), right insula (Insula), right amygdala (Amyg), and left middle temporal gyrus (MTG)], functionally defined frontal eye fields (FEF), motion-sensitive area V5 (V5), primary visual cortex V1 (V1), and anatomically defined superior parietal lobule (SPL). Connectivity for MdDS is shown in red; Controls (Ctrl) in blue. Connectivity differences of anterior to posterior nodes between MdDS and Ctrl participants are shown pictorially in **(B)**. Figure adapted from Cha YH et al., *PLOS One* 2012 (4).

Comparisons between individuals with MdDS and healthy controls using this seed-based analysis revealed two distinct patterns of connectivity differences. First, individuals with MdDS showed decreased connectivity between the entorhinal cortex/amygdala seed and the left superior medial gyrus, left middle frontal gyrus, left superior and middle temporal gyri, FEF, and right insula, while showing increased connectivity with areas V1, V5, SPL, middle temporal gyrus, and right amygdala (Figure 2A). The general pattern was of increased connectivity with parieto-occipital visual–vestibular areas but reduced connectivity with prefrontal and temporal areas (Figure 2B). Second, functional connectivity was reduced between all homologous brain regions queried except for areas V1 and V5 in which the interhemispheric connectivity between MdDS and controls participants was the same. One interpretation was that this pattern represents enhanced visuo-spatial information transduction to a core hub of spatial information processing, which is under less regulatory control. Activation of the entorhinal cortex and amygdala neurons inhibits prefrontal neurons and vice versa, which are components of a network that can act in a reverberating manner (46–48). A mutually inhibitory process could lead to a “winner take all” situation and be subject to toggling from one control system to another. These connectivity differences do not indicate whether the effects are primary or secondary or which hub of the network is critical for persistence of symptoms, however.

**Figure 3:**
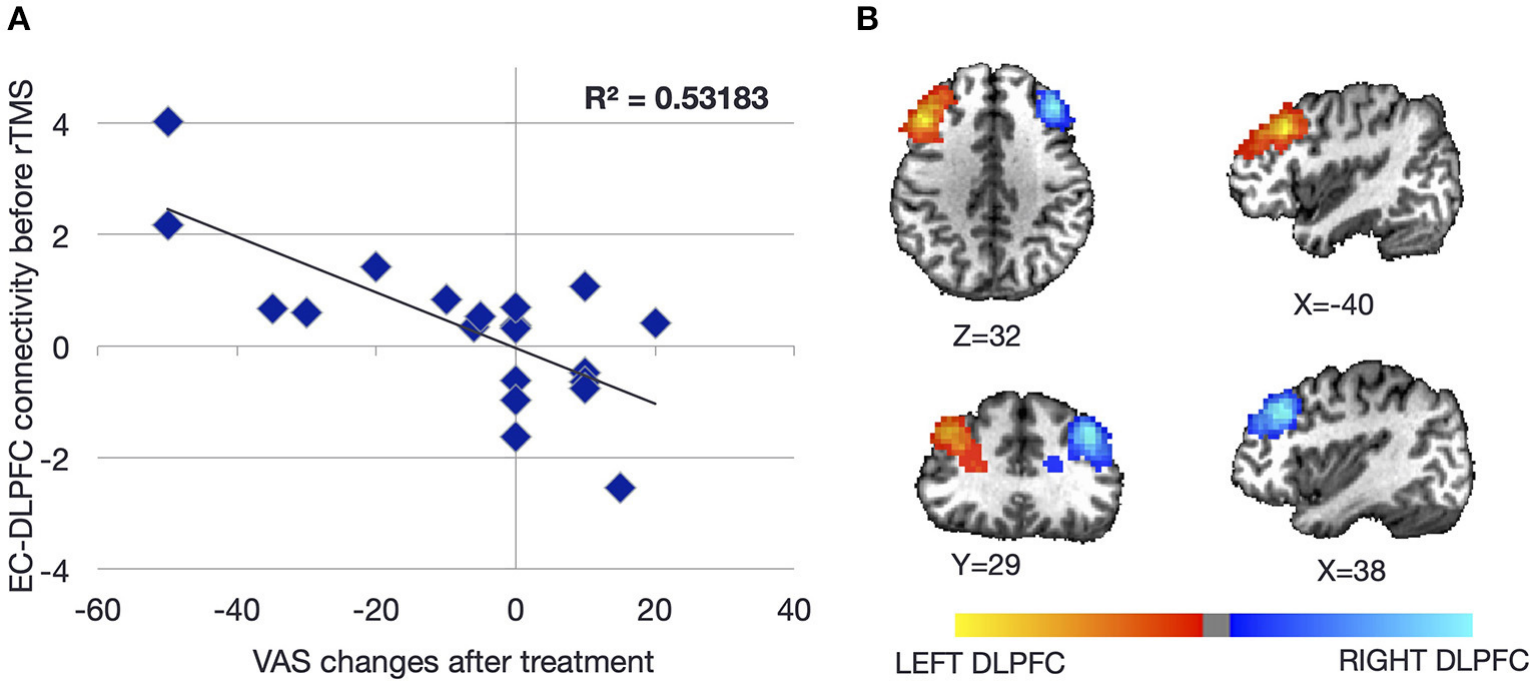
Baseline resting-state functional connectivity between the left and right dorsolateral prefrontal cortices (DLPFCs) and the ipsilateral entorhinal cortex as a function of symptom severity change after repetitive transcranial magnetic stimulation (rTMS) to the DLPFC. Individuals with higher baseline connectivity between the DLPFC and ipsilateral entorhinal cortex responded better to rTMS **(A)**. The location of each DLPFC was determined from individualized neuronavigation targets aiming for the anterior portion of the middle of the middle frontal gyrus **(B)**. Coordinates of the entorhinal cortex were determined from individual structural scans. Figure adapted from Yuan et al., *Brain Connectivity* 2017 (49).

Change in functional connectivity measured by fMRI between the entorhinal cortex/amygdala and the whole brain as a function of response to transcranial magnetic stimulation (TMS) was assessed in 20 right-handed women who underwent five sessions of prefrontal repetitive TMS (rTMS) [mean age 52.9 (range of 28–68 years), median symptom duration of 30 months (range of 8–96 months)] (49). Symptom severity was measured on a 100-point visual analog scale with symptom change measured as a categorical variable of “positive,” “neutral,” or “negative,” depending on whether symptoms improved, stayed the same, or worsened, respectively. Decreasing scores represented decrease in symptoms. The specific treatment entailed 1,200 pulses over the right DLPFC at 110% of the motor threshold (MT), followed by 2,000 pulses over the left DLPFC at 110% MT. Pearson's correlation coefficients were calculated between seeds in the left and right entorhinal cortices (defined in individual brains) (Figure 3A) and the specific locations in the DLPFC (Figure 3B) that were stimulated with neuronavigation (Localite^®^) guidance. The goal was to determine resting-state functional connectivity change as a function of symptom change as well as baseline measures of connectivity that correlated with treatment response.

**Figure 4:**
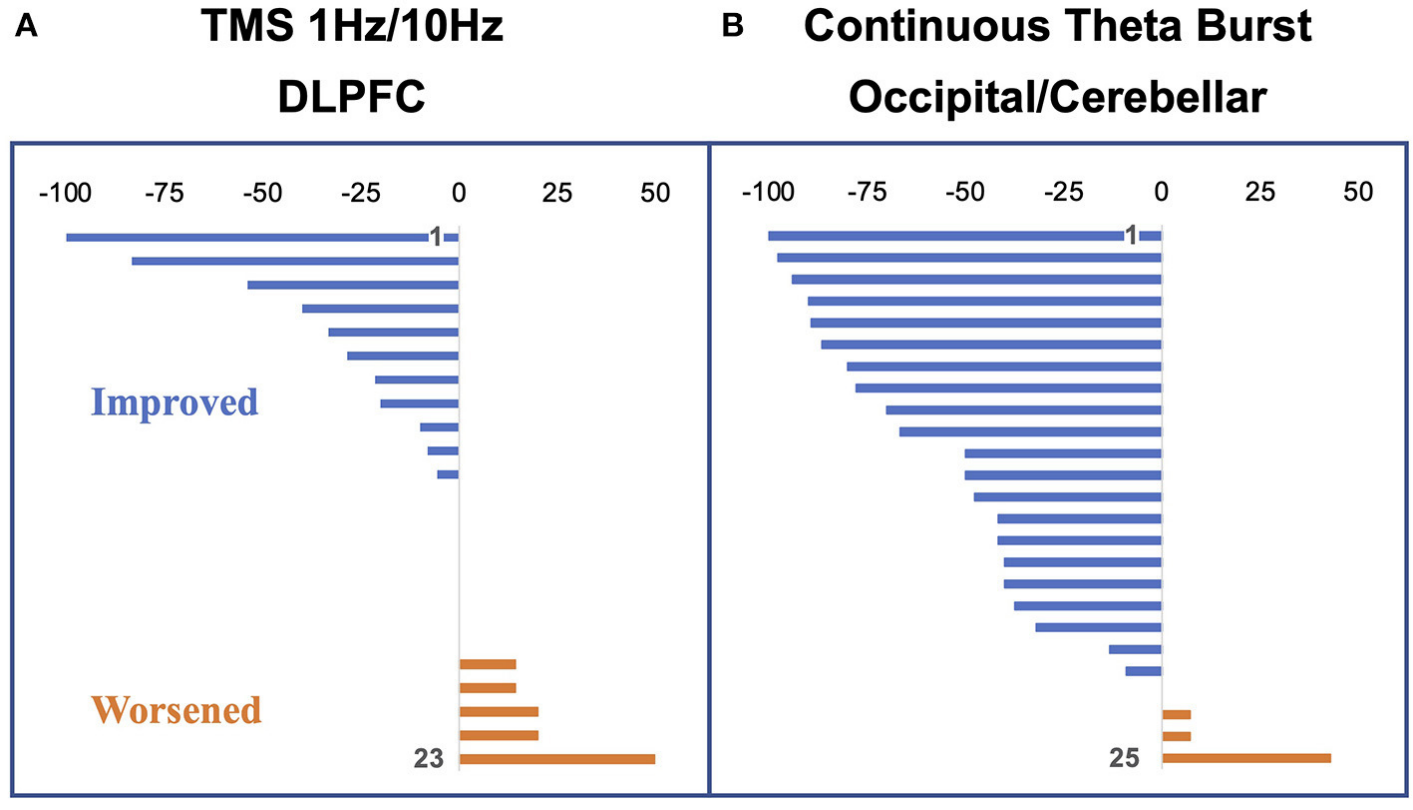
Percent change in vertigo intensity measured as a visual analog scale change from pre to post stimulation. Negative deflections represent a decrease in symptoms; positive deflections, an increase in symptoms. **(A)** Participants in the dorsolateral prefrontal cortex (DLPFC) repetitive transcranial magnetic stimulation (rTMS) study (24 at baseline with one dropout). **(B)** Participants in the occipital/cerebellar theta burst study (26 participants with one dropout). Figure adapted from Cha YH et al., *Brain Stimulation* 2016 (50). and Cha YH et al., *Otology Neurotology* 2019 (51).

Of the 20 individuals, six reported improvement, eight reported no significant change, and six reported worsening using a cutoff of 10 points on the visual analog scale (Figure 4A). Notably, the degree of improvement was much greater than the degree of worsening such that the response of the “neutral” group was similar to that of the “negative” group. In a conjunction analysis that required symptom and connectivity changes to the entorhinal cortex to correlate in a bidirectional manner, i.e., decrease with improvement and increase with worsening or vice versa, three brain regions were identified. Decrease in resting-state functional connectivity between the left entorhinal cortex and right IPL, left precuneus, and right entorhinal cortex correlated with symptom improvement, while increases in connectivity correlated with no change or symptom worsening (49). Corresponding to the closer scoring change between the neutral group and the negative response (worsening) group compared with the positive response (improvement) group, the connectivity changes were similar between the neutral and negative response groups. High baseline connectivity between the entorhinal cortex and the ipsilateral DLPFC correlated with response to rTMS in a continuous manner, perhaps indicating that a higher dynamic range of potential modulation is critical for treatment response (Figure 3A).

This study was one of the first to show that symptom improvement in MdDS corresponds to a general decrease in functional connectivity between posterior sensory processing regions and the entorhinal cortex. The higher baseline connectivity between posterior parietal and occipital cortex with the entorhinal cortex in individuals with MdDS compared with healthy controls may have been reversed in treatment responders (4). These data suggest that one goal of therapy may be to reduce parieto-occipital to limbic connectivity.

## Structural Magnetic Resonance Imaging

Structural brain characteristics of MdDS were assessed with voxel-based morphometry (VBM). VBM is an imaging tool used to make voxel-by-voxel comparisons between segmented brain tissues (52). These methods can reveal subtle differences in regional brain volume that cannot be detected with clinical imaging. Whereas a typical T1 structural image acquired for clinical purposes may include 36 slices for the whole brain, a T1 structural image used for VBM analysis may use 180+ slices. This allows the brain volume to be segmented into gray matter, white matter, and fluid volumes at a resolution of 1 mm^3^. VBM analysis was performed on 28 individuals with MdDS compared with 18 healthy age- and sex-matched controls (53). Individuals with MdDS had a mean age of 43.0 ± 10.2 years and median duration of illness of 24 months (range 3–240 months). Without the outlier of 240 months, the mean duration was 35.7 ± 28.7 months.

In head-to-head whole brain contrasts, MdDS participants exhibited increased volumes in the following areas: the left IPL, right ventral occipital lobe (V3v), and right temporal lobe in the cerebrum and in bilateral hemispheric lobules VIIIb and IX, left Crus I, VIIa/VIIb, and VIIIa in the cerebellum (*t* > 3.0, *p* < 0.005_uncorr_). Decreased volumes relative to controls were noted in the following areas: the bilateral middle orbital gyri, left pgACC, left middle temporal gyrus, left calcarine gyrus, and right inferior frontal gyrus (IFG) in the cerebrum and the left cerebellar lobule VIIa Crus II in the cerebellum. These analyses were done with nuisance covariates of age, and the anxiety and depression subscores of the HADS. Specific interrogation of motion-sensitive area V5/MT and the parieto-insular vestibular cortex (PIVC) were assessed because both regions receive vestibular projections and show volume changes after peripheral vestibular injury (54, 55). No volume differences were found between MdDS participants and controls in direct contrasts within these regions, however.

**Figure 5:**
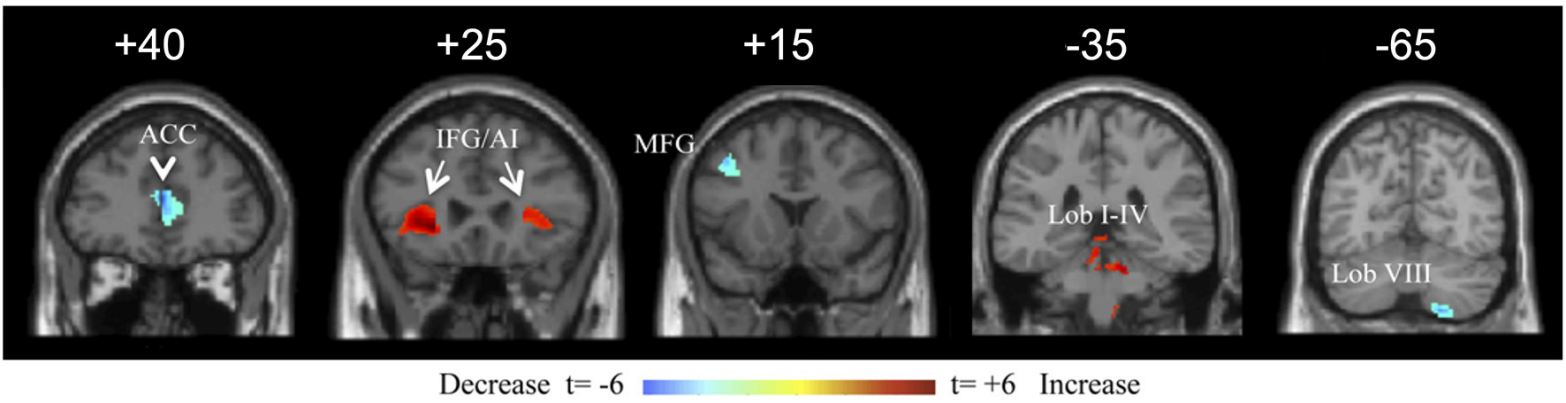
Coronal view of a selection of brain regions with volume changes as a function of duration of illness of mal de débarquement syndrome (MdDS). Blue indicates volume decrease with duration of illness; red indicates volume increase with duration of illness. Coordinates of peak significance are indicated in parentheses. In SPM, negative *X*-values are on the left and positive on the right. ACC, anterior cingulate cortex [Montreal Neurological Institute (MNI): ±2, 40, 13). IFG/AI: inferior frontal gyrus/anterior insula (MNI: −26, 30, −3 and 27, 24, 4). MFG: middle frontal gyrus (MNI: −42, 15, 43). Lob I–IV: cerebellar lobules I–IV (MNI: −6, −38, −17 and 8, −50, −27). Lob VIII: cerebellar lobule VIII (18, −66, −55). Images are shown at the Y coordinate indicated above each image. Figure adapted from Cha YH and Chakrapani S, *PLOS One* 2015 (53).

Multiple regression analysis for duration of illness showed two prominent cerebral areas with duration sensitive volume changes at a false discovery rate < 0.05. The pgACC volume decreased with duration with a correlation coefficient of −0.633 (*p* < 0.05_corr_). Both IFG/anterior insular (AI) regions increased in volume with duration, with left and right correlation coefficients of +0.440 and +0.427, respectively (*p* < 0.05_corr_) (Figure 5). Other cerebral areas that showed positive correlation with duration at a lower significance (*t* > 3.0, *p* < 0.005_uncorr_) included the bilateral postcentral gyri, left superior occipital gyrus (V3a), right Heschl's gyrus, right cuneus, left middle occipital gyrus (V5/MT), left amygdala, left SPL, left lingual gyrus (V3v/V4v), bilateral calcarine gyri, left middle and inferior temporal gyri, and right temporal pole. In the cerebellum, the bilateral anterior cerebellum (lobules I–IV), left hemispheric lobule IX, and vermian lobule IX (uvula) increased in volume with duration. Cerebral volume decreases with duration included the bilateral middle ACC and the left middle frontal gyrus (area of the DLPFC). The right cerebellar lobule VIIIa/b was the only cerebellar region to decrease in volume with duration of illness (Figure 5). As a caveat, the volume changes in this study were measured in a cross-sectional rather than longitudinal manner. Therefore, whether they would show the same changes within an individual is not determined.

The most significant volume-related markers of MdDS were correlations of decreased pgACC volume and increased IFG/AI volumes with duration of illness. The pgACC is functionally connected to the limbic system, specifically reducing amygdala activity and activating in tasks related to emotional conflict regulation, fear extinction, and planning of responses to threats (56–59). The lower volume in this region as a function of longer duration of illness may reflect less limbic regulatory control as either a cause or a result of prolonged symptoms. The IFG/AI is a major hub of the SN (along with the dorsal ACC, amygdala, ventral striatum, and ventral tegmental area/substantia nigra), a network that is activated when assessing functionally relevant stimuli (60). Duration-related volume changes in the threat regulation system and the SN could relate to decreased ability to appraise potential environmental threats along with increased demand on filtering sensory stimuli. Dysfunction within these networks could contribute to prolonged symptoms and increased morbidity. Reduced performance of the SN's function in toggling between the DMN and ECN could lead to an inability to efficiently activate working memory networks or lead to heightened interoceptive awareness (60, 61). This could potentially contribute to the problem of cognitive dysfunction (“brain fog”) frequently reported by individuals with MdDS in which it is difficult for them to focus on task-relevant stimuli (2, 33).

A role for the cerebellum in MdDS may be related as much to its connectivity with resting-state networks as it does to specific pathways within the vestibular system. Bilateral cerebellar lobules VIII/IX showed increased volume compared with controls. Lobule VIII is functionally connected to the premotor cortex and is thus relevant for the planning of motor movements (38, 62). The right cerebellar lobule VIII, which was the only cerebellar region to show a negative correlation with duration of illness, is functionally connected to every portion of the precuneus, a hub of the DMN that plays a critical role in memory, attention, and visuospatial processing (63, 64). Cerebellar lobule IX (uvula) is functionally connected to the DMN but also receives ipsilateral primary vestibular afferents and bilateral secondary vestibular afferents in a complex with lobule X (nodulus) (65). There has been no peripheral vestibular injury noted in MdDS to date, but a hypothesis that MdDS pathophysiology could be related to a prolonged vestibulo-ocular reflex time constant that can be readapted using optokinetic stripes could be related to posterior cerebellar volume changes (66). Therefore, multiple potential hubs of the DMN and a hub for vestibular processing may be affected in MdDS.

## Electroencephalography

A case was reported of a marker of transient MdDS using an EEG-based source localization method called standardized low-resolution brain electromagnetic tomography (sLORETA) (67). sLORETA is a method for localizing electrical activity within the brain by the use of surface electrodes on the scalp (68). A 20-year-old man who experienced a swaying sensation and dizziness after a boat trip and bus ride was evaluated during the symptomatic period 3 days after the onset and after resolution of his symptoms 10 days later. It was reported that compared with resolution, the patient's brain in the symptomatic period exhibited a decrease in alpha (8–12 Hz) power in the left precentral gyrus and an increase in beta-2 power (19–21 Hz) in the right para-hippocampal region. With the caveat that sLORETA has poor localization in deep brain regions, this report highlights the potential role of structures involved in spatial navigation in MdDS symptomatology.

**Figure 6:**
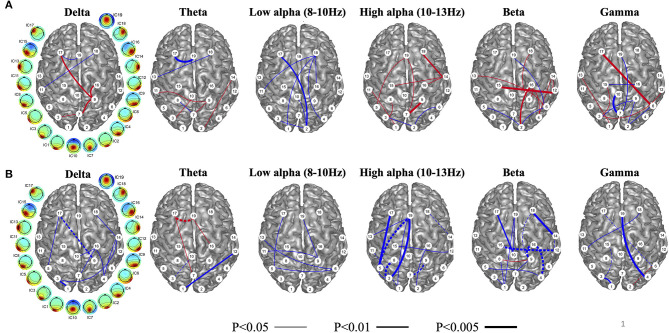
Independent component phase coherence (ICPC) changes reflected as increases (blue) and decreases (red) of ICPC as a function of treatment response to repetitive transcranial magnetic stimulation (rTMS) over the dorsolateral prefrontal cortex (DLPFC) **(A)**. As a function of treatment response, connectivity in the high alpha, beta, and gamma, and delta bands decrease, while connectivity in the low alpha band increases with treatment response. Changes in the theta frequency are mixed. Baseline connectivity as a function of treatment response (blue represents high, and red represents low) in **(B)**. Baseline connectivity is high across all frequency bands in treatment responders. Lines connecting independent components are weighted for higher statistical significance. Dashed lines indicate connectivity that both changes and predicts treatment response. Figure adapted from Cha YH et al., *Brain Connectivity* 2018 (70).

In the same studies that evaluated rTMS over DLPFC, functional connectivity with high density EEG was recorded before and after the intervention. Measurement of this connectivity was reflected through independent component phase coherence (ICPC), a calculation of synchronicity at individual frequency bands (delta, theta, low alpha, high alpha, beta, and gamma) on an independent component level rather than an individual channel level (69). ICPC calculations on 128-channel EEG data collected pre and post rTMS have shown distinct differences in synchronizations across independent component regions and frequencies (69, 70). In general, reduction in ICPC in the delta, high alpha, beta, and gamma bands were noted with symptom improvement, whereas increase in ICPC was noted in the low alpha band (Figure 6A). Most desynchronizations were either long-range frontal-parieto-occipital or parieto-occipital with some connectivity crossing hemispheres.

Similar to fMRI analyses, baseline high ICPC values correlated with greater treatment response; high ICPC values across all frequency bands portended better treatment response (Figure 6B). This may, again, correlate with a higher dynamic range of potential desynchronization being possible in individuals with higher baseline connectivity. The magnitude of treatment response was related to the magnitude of the number of ICPC pairs that desynchronized, i.e., more desynchronized ICPC pairs correlated with greater symptom reduction, suggesting that the general goal of treatment may be to induce more desynchronization and particularly at the high alpha and beta frequencies.

In addition to the above ICPC studies that have revealed biomarker information at the EEG sensor level, investigations of EEG source level computations have been able to reproduce resting-state networks that spatially correspond to the standard template of brain networks (71–73). Our data revealed that EEG source network changes in the left medial frontal gyrus and primary visual cortex positively correlate with symptom changes, whereas EEG source network changes in the right middle temporal gyrus negatively correlate with symptom changes (72, 73). Furthermore, baseline EEG connectivity values in the primary visual cortex were found to predict symptom changes induced by rTMS, with particularly high baseline connectivity predictive of reduction of symptoms. In the visual cortex, functional connectivity decreased after rTMS in five out of six positive responders, notable since the visual cortex had previously been shown to exhibit higher baseline connectivity with the entorhinal cortex in MdDS (4). To date, EEG source imaging findings have corroborated symptom-related dynamic changes in fMRI, converging on the theory that the therapeutic mechanism of rTMS is to normalize pathological connectivity in MdDS.

## Simultaneous Functional Magnetic Resonance Imaging–Electroencephalography

Simultaneously acquired fMRI-EEG data on individuals with MdDS have provided cross-modal validation of biomarkers found separately, showing that brain network-level changes associated with clinical effects are consistent across modalities of different temporal resolutions (49, 69, 70, 72–74). Our data showed that after rTMS at DLPFC, EEG synchronization changes in medial frontal gyrus were associated with fMRI-measured connectivity changes involving deeper cortical structures, particularly in a network that includes the entorhinal cortex and right IPL, indicating that the modulatory effect of rTMS is at least partially related to reducing the connectivity within the DMN (49, 73). Such a mechanism appears to be consistent with findings suggested in other disorders treated by rTMS over DLPFC, such as major depression (75, 76).

Our multimodal imaging data of EEG and fMRI have shown that improvement in symptom severity was correlated with a reduction in connectivity involving multiples nodes of the DMN, including the entorhinal cortex, precuneus, IPLs, and medial frontal gyrus. Specifically, in the positive responders to rTMS, a significant reduction of symptoms was associated with a reduction in connectivity in the medial frontal gyrus—a key node of the DMN. In the DLPFC stimulation protocol, we have shown that the stimulation did not lead to increased connectivity between the stimulation site and entorhinal cortex itself, however, as we had hypothesized based on lower prefrontal-to-limbic connectivity in MdDS (4, 49). Rather, our data showed that stimulation at DLPFC played a modulatory role, resulting in decreases of connectivity between posterior DMN and the entorhinal cortex (49). Such a phenomenon of decreased connectivity in DMN nodes associated with symptom reduction has been observed in a later study utilizing continuous theta burst stimulation (cTBS) over the occipital cortex and cerebellar vermis and interrogating connectivity with EEG (51, 77). While the DLPFC rTMS protocol yielded 3 out 23 participants experiencing ≥50% symptom reduction, the cTBS over occipital cortex and cerebellar vermis protocol yielded 12 out of 25 participants with ≥50% symptom reduction (50, 51) (Figure 4B). This improved efficacy may be related to more direct engagement of fronto-parieto-occipital connectivity (both through the occipital cortex target and through functional connectivity through the vermis), greater treatment numbers, and more direct entrainment effects (42, 78–80).

Combined EEG-fMRI studies have suggested a strategy of using multimodal data to guide rTMS in MdDS. These investigations have indicated that connectivity within the DMN, measured by fMRI as well as EEG, can be an imaging-based symptom biomarker. Treatments guided by the modulation of this biomarker may be informative in trials of brain stimulation, potentially not limited to rTMS. More importantly, although fMRI reveals symptom-related connectivity, an EEG-based targeting strategy would be compatible with simultaneous rTMS and may provide instantaneous feedback in trial sessions of stimulation protocols. Biomarkers from the two modalities can be integrated to guide rTMS targets. For example, the high spatial resolution of fMRI can be used to capture a disease-modifying network involving deep cortical or subcortical structures, whereas connectivity involving superficial nodes of these networks can be identified with EEG, which has high temporal resolution, potentially through a matching procedure with the fMRI-measured network as described in our approaches. New stimulation protocols could therefore be designed to promote network modulation in desired directions.

## Conclusion

MdDS represents a model of the human brain's entrainment to motion that results in a persistent sense of oscillating vertigo. The anatomical substrates for this perception have been interrogated with multiple neuroimaging modalities in order to evaluate metabolism, functional connectivity, brain volume, and synchronicity. A developing model for MdDS hypothesizes that at least one hub of the neural network that contributes to persistence of symptoms includes the left entorhinal cortex and amygdala. Through their reciprocal inhibitory action on the prefrontal cortex, these structures may wield outsized effects on both task-specific and amodal resting-state networks. Symptoms that are common in MdDS such as cognitive difficulty and feeling of sensory overload might be attributed to inefficiencies in toggling between resting states, e.g., DMN and ECN, and in dysfunction in neural substrates that filter irrelevant sensory stimuli, e.g., SN. A general neuroimaging feature of improved symptoms appears to be desynchronization of medium- and long-range connections, particularly between the parieto-occipital cortex and limbic areas as well as between frontal and parieto-occipital cortices. As further probing of these connections occurs through modulating symptoms with noninvasive brain stimulation, a more detailed understanding of the core neural components that drive persistence MdDS should emerge.

## Author Contributions

YC, LD, and HY were all involved in the manuscript preparation, figure generation, and review process. All authors contributed to the article and approved the submitted version.

## Conflict of Interest

The authors declare that the research was conducted in the absence of any commercial or financial relationships that could be construed as a potential conflict of interest.
